# A restraint molecular dynamics and simulated annealing approach for protein homology modeling utilizing mean angles

**DOI:** 10.1186/1471-2105-6-91

**Published:** 2005-04-08

**Authors:** Andreas Möglich, Daniel Weinfurtner, Till Maurer, Wolfram Gronwald, Hans Robert Kalbitzer

**Affiliations:** 1 Institut für Biophysik und physikalische Biochemie, Universität Regensburg, Universitätsstr. 31, D-93053 Regensburg, Germany; 2 Department of Biophysical Chemistry, Biozentrum, University of Basel, Klingelbergstr. 70, CH-4056 Basel, Switzerland; 3 Institut für Organische Chemie und Biochemie, Technische Universität München, Lichtenbergstr. 4, D-85747 Garching, Germany; 4 Department of Lead Discovery, Boehringer Ingelheim Pharma GmbH, Birkendorfer Str. 65, D-88397 Biberach, Germany

## Abstract

**Background:**

We have developed the program PERMOL for semi-automated homology modeling of proteins. It is based on restrained molecular dynamics using a simulated annealing protocol in torsion angle space. As main restraints defining the optimal local geometry of the structure weighted mean dihedral angles and their standard deviations are used which are calculated with an algorithm described earlier by Döker *et al*. (1999, *BBRC*, **257**, 348–350). The overall long-range contacts are established via a small number of distance restraints between atoms involved in hydrogen bonds and backbone atoms of conserved residues. Employing the restraints generated by PERMOL three-dimensional structures are obtained using standard molecular dynamics programs such as DYANA or CNS.

**Results:**

To test this modeling approach it has been used for predicting the structure of the histidine-containing phosphocarrier protein HPr from *E. coli *and the structure of the human peroxisome proliferator activated receptor *γ *(Ppar *γ*). The divergence between the modeled HPr and the previously determined X-ray structure was comparable to the divergence between the X-ray structure and the published NMR structure. The modeled structure of Ppar *γ *was also very close to the previously solved X-ray structure with an RMSD of 0.262 nm for the backbone atoms.

**Conclusion:**

In summary, we present a new method for homology modeling capable of producing high-quality structure models. An advantage of the method is that it can be used in combination with incomplete NMR data to obtain reasonable structure models in accordance with the experimental data.

## Background

Due to the enormous progress that has been made in genomics a large number of DNA sequences including many whole genomes have been published. The evaluation of these data must include the determination of the three-dimensional structures of the proteins encoded. Although the two experimental techniques capable of determining three-dimensional structures of proteins and other biomolecules at atomic resolution, namely nuclear magnetic resonance (NMR) and X-ray crystallography, have seen significant improvements the process of structure determination remains very time-consuming and difficult. Unless unexpected advances of these techniques will occur in future, it is obvious that for the majority of all the primary sequence data available three-dimensional structures cannot be obtained experimentally. Therefore, only computational approaches are capable of filling the gap between existing protein sequences and structures. Although considerable progress has been achieved in *ab initio *structural prediction strategies [1-3] they are in general still unreliable when atomic resolution is demanded. However, when structures of homologous proteins are available, the prediction of the three-dimensional structure of entire proteins and protein domains is rather successful. In light of the fact that the protein structures elucidated so far only show a remarkably limited number of folds it would be desirable to accelerate the structure determination process especially for proteins possessing a fold already known. According to the SCOP classification [4,5] (release 1.65, 1 August 2003) 20619 protein structures stored in the Protein Data Bank share only 800 different folds. Comparison of different proteins with similar amino acid sequences showed that they quite often display very similar tertiary structures [6-9].

In the past several different homology modeling approaches were published which range from strongly interactive methods (model building) to fully automated methods (for reviews see e. g. [10] and [11]). Generally the starting point in these approaches is a search in structure databases such as the Protein Data Bank [12] or CATH [13] for all protein structures that are related to the target sequence and then to select those 3D structures that will be used as templates. For searching the structural databases one can employ pairwise sequence-sequence comparisons using for example programs such as FASTA [14] and BLAST [15]. When increased search sensitivity or a larger number of homologs are demanded methods which are based on multiple sequence alignments prove to be particularly efficient. Such an algorithm is implemented in the program PSI-BLAST [16]. An alternative strategy for homolog identification relies on so-called threading methods, which predict whether the target sequence adopts any of the known 3D folds. Threading methods should be useful in cases when no sequences can be found which are clearly related to the target [17].

When a list of related protein structures has been obtained the appropriate templates have to be chosen from these. In this procedure usually factors such as high overall sequence similarity between target and template sequences, quality of the template structure and conditions under which the template structure was obtained are taken into account. Then the selected templates have to be optimally aligned with the target sequence. Since the search methods mentioned above are usually optimized for detecting remote homologs they are not optimal for target-template alignment. A program often used for the latter type of alignments is CLUSTALX [18], which is also used within PERMOL.

Using the template-target alignment a variety of methods has been published for 3D model building. The group of methods which were developed first and are still frequently used were modeling by rigid body assembly [19-21]. Another group of methods use segment matching [22-25]. In the third, most recent group of methods spatial restraints obtained from the template structures are used in distance geometry calculations or energy optimization procedures to obtain the target model [26-31].

The PERMOL approach described presently also uses spatial restraints but in contrast to most other programs mainly dihedral angle restraints as opposed to restraints derived from inter-atomic distances are employed. These restraints enter molecular dynamics calculations in torsion angle space. In the following we will describe this method in more detail and mark differences to existing programs that have been published before.

In *ab initio *molecular dynamics (MD) simulations in addition to the applied force field only information about the amino acid sequence of the protein in study enters the calculations. While for small molecules such methods show results that are in very good agreement with the experimental data they mostly fail for more complex molecules. On the other hand restrained molecular dynamics calculations based on simulated annealing protocols are routinely and successfully used for the determination of solution NMR structures – in that case strong experimental information is available. Especially effective with regard to computational effort are calculations in torsion angle space as implemented in the programs DYANA [32] and CNS [33].

In this contribution we propose a method which combines the well-developed torsion angle dynamics calculations of DYANA or CNS with structural information extracted from three-dimensional structures of homologous proteins. This information is translated into conformational restraints. Local structural restraints are obtained by a weighted average of the backbone dihedral angles using an algorithm proposed by Döker *et al*. [34] These averaged dihedral angle restraints are usually well preserved within the local secondary structure elements and therefore are especially well suited for the modeling of these. The program MODELLER [28] for example also uses dihedral angle restraints in an optimization procedure but expresses them as so-called probability density functions which are derived from structural features in several families of homologous proteins. Global structural restraints are obtained from distance relations between carefully selected atoms of amino acids well separated in the primary structure. In contrast to other programs the distance restraints are mainly used for the global arrangement of the secondary structure elements which are defined by the dihedral angle restraints. The efficient structure calculations performed with DYANA or CNS allow calculating a large number of structural models in a relatively short amount of time. From the resulting ensemble of structures the best in terms of the DYANA target function or total energy (CNS) can be selected for further analysis. As has also been shown in NMR spectroscopy, it is useful to describe the target structure by an ensemble of model structures.

It should be noted that the PERMOL approach described here is related to the method detailed by Zhang *et al*. [35], which uses a combination of torsion angle dynamics and dihedral angle and distance restraints to predict the fold of helical proteins. In contrast to PERMOL the program from Zhang *et al*. uses methods for secondary structure and contact prediction to derive spatial restraints.

To benchmark the PERMOL approach we used it to determine a homology structure for the histidine-containing phosphocarrier protein (HPr) from *E. coli *of which the structure has been solved experimentally both by NMR [36] (PDB entry: 1HDN) and X-ray crystallography [37] (1POH). The homology model was compared to the target structures and to a homology model calculated with the program MODELLER [28]. To also investigate the performance of PERMOL on larger proteins that contain substantial disordered loop regions the human peroxisome proliferator activated receptor *γ *(Ppar *γ*) was used as a test case. Its structure has been determined previously by X-ray crystallography [38] (3PRG).

## Results

### Theoretical considerations and general strategy

In standard NMR structure determination the principal physical model of a protein is represented by empirical potentials determining the general geometry. The fast optimization is obtained by a simulated annealing protocol and the correct conformations are selected from the generally accessible conformational space by the experimental restraints which are transformed into pseudo-potentials. In the approach used in PERMOL the experimental restraints are replaced by restraints derived from three-dimensional structures of homologous proteins. Local conformations are optimally encoded by the distribution of the corresponding torsion angles. The overall fold is determined by distance relations since even small errors in dihedral angles can add up to very large distance errors between amino acids that are separated by several positions in the sequence.

The use of a molecular dynamics and simulated annealing protocol for homology modeling allows to encode the features of the statistical distribution of a given parameter *α*_i _individually for each group of restraints. To this end not only the expectation values  are calculated from the homologous structures *j *(*j *= 1,..,*N*_*i*_) but also the upper and lower limits,  and . It is still under discussion in the NMR community how exactly the upper and lower limits of restraints have to be defined but it is clear that they are related to the expected error of a given, individual parameter. A generally accepted definition is not available yet. In addition the form of the pseudo-energy function used in the calculations has to depend on the error distribution of the given parameter (see e. g. [39]).

The homology modeling procedure proposed here comprises the following steps: step 1, selection of data and sequence alignment, step 2, selection of restraints, and step 3, the restrained molecular dynamics simulation. These conceptually different steps in the calculation are reflected in the implementation of PERMOL in corresponding levels of the modeling procedure.

### Level 1 – Selection of data and sequence alignment

Initially, one or several structures of homologous proteins are selected as templates. Their amino acid sequences are aligned to the sequence of the target protein using the program CLUSTALX [18]. The resulting alignment is written to a text file and can be edited by the user. Conserved amino acids are characterized and classified for manual or automated selection of restraints. Based on the degree of sequence conservation in the different proteins a homology score value *v*_i _is calculated for each residue. The score values *v*_i _range from 1.0 for a completely conserved residue to 0.1 for a residue, which in the template proteins has been replaced in a non-conservative manner, e.g. a hydrophobic residue replaced by a charged one.

### Level 2 – Selection of restraints

For the calculation of dihedral angle restraints usually only Φ and Ψ angles are taken into account but the *ω*-angle can be included as well. Structural restraints are only derived from residues, which have been selected. Additional residues can be selected either manually or automatically based upon the score value *v*_i_. Expectation values and standard deviations are calculated as described in the 'Methods' section with  set to 1/ when  structures are found in the pdb-file k as it is often the case for NMR-structures. Upper and lower limits for the dihedral angle restraints can be calculated either as the mean value plus/minus multiples of the standard deviations, <*α*_i_> ± b* <*s*_i_> with a user defined constant *b*, or as the mean angle plus/minus a constant value. An additional weighting of the individual restraints can be performed on the basis of the score value *v*_i _which modifies the force constant of the restraint i in the MD calculation.

By default, distance restraints are automatically computed between the N^H ^atoms of completely conserved amino acids. Restraints can also be generated for additional amino acids and atom types by appropriate selection. For the generation of distance restraints similar options are possible as for dihedral angle restraints. In addition, an upper distance limit for the pairs of atoms to be considered can be defined.

Conserved hydrogen bonds can also be used to generate distance restraints between the atoms involved in forming the bond. The criteria for selecting hydrogen bonds in the homologous protein structures can be modified by the user. By default, only hydrogen bonds are considered for which the N-O distance does not exceed 0.24 nm and the angle between the N^H^-H^N ^and the C = O bond vectors does not deviate by more than 35° from 180°. Again, different options are possible for the calculation of the upper and lower limits. Hydrogen bonds which occur only in a few structures or are assigned to more than one pair of atoms, e.g. due to deviations between the different homologous proteins used as templates, can be automatically removed by corresponding filter functions.

### Level 3 – Restrained molecular dynamics simulation

The restraint files generated by PERMOL can be directly used by the molecular dynamics programs DYANA and CNS. Standard simulated annealing protocols are employed.

### Modeling of HPr from E. coli and of human Ppar *γ*

To test the modeling approach described in this paper we determined a homology structure for the histidine-containing phosphocarrier protein (HPr) from *E. coli*. HPr is an integral part of the bacterial phosphoenolpyruvate dependent phosphotransferase system (PTS) which efficiently catalyses phosphorylation and the import of carbohydrates into prokaryotic cells [40]. HPr molecules from different organisms have been extensively studied and many 3D structures have been elucidated. In particular the structure of HPr from *E. coli *has been solved both by NMR [36] (PDB entry: 1HDN) and X-ray crystallography [37] (1POH) and is thus especially suited to test our modeling strategy (see Table 1).

Four previously determined HPr structures from four different organisms have been used as model structures (PDB codes 1PTF, 1QFR, 1QR5, and 2HID). An overview of these structures is given in Table 1. Only 21 % of the amino acid sequence is strictly conserved between the HPr proteins of *E. coli*, *S. faecalis*, *E. faecalis*, *S. carnosus*, and *B. subtilis *(18 out of 85 residues). Spatial restraints for the structure calculation were generated as detailed in the 'Methods' section. For the derivation of inter-atomic distance restraints only residues which are completely conserved or display conservative amino acid exchanges (e. g. one hydrophobic residue replaced by another one) were considered. Upper and lower limits for these distances were determined as the mean distance value plus or minus the standard deviation, respectively. Restraints for the backbone dihedral angles Φ and Ψ were calculated for all residues and have been weighted according to the homology score value *v*_i_. Upper and lower limits were determined as for the distance restraints. Hydrogen bonds were analyzed using the default parameter values. Distance restraints between the corresponding H^N ^and O atoms were computed as the mean distance value plus or minus the standard deviation. A summary of these restraints is presented in Table 3.

**Figure 1:**
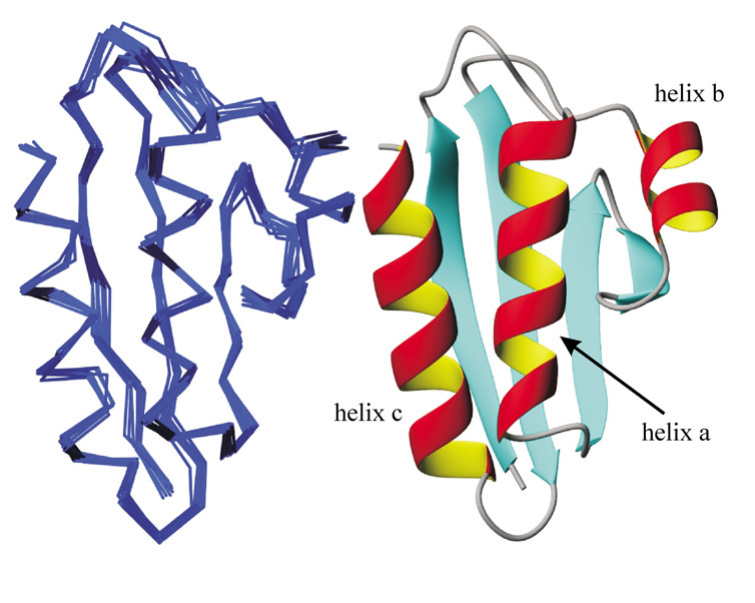
*Homology structures of HPr from E. coli determined by PERMOL*. Ensemble of the 10 homology structures with the lowest pseudo-energy out of 200 structures calculated with DYANA. (left) A superimposition of the C^*α *^atom traces is shown. (right) A cartoon representation of the mean structure of the 10 models is displayed.

Based on these restraints an ensemble of homology structures was computed using the molecular dynamics program DYANA [B32] with the standard simulated annealing protocol. Out of 200 structures calculated, the group of the ten structures with the lowest pseudo-energies was further analyzed. These ten models showed a good convergence with a RMSD value for the backbone atom positions of 0.041 nm (Fig. 1, Table 5). They displayed the well-known secondary structure elements common to all HPr molecules studied so far, comprising a four-stranded antiparallel *β*-sheet and three *α*-helices designated as helices a, b, and c. Analysis of the ensemble of these ten structures with PROCHECK-NMR [41] showed that all backbone dihedral angles fell into the most favored and additionally allowed regions of the Ramachandran plot (Table 5). Modeling experiments where the dihedral angle restraints have been partly or completely left out from the structure calculations of the model structures underlined their importance in defining the correct secondary structure and local conformations (see below).

In order to further test our modeling strategy we set out to derive a homology structure for the human peroxisome proliferator activated receptor *γ *(Ppar *γ*). Ppar *γ *is considerably larger than HPr and comprises about 280 amino acid residues. Further, it contains larger relatively unstructured loop regions and it is worthwhile to investigate how PERMOL performs here. In addition this molecule is of particular importance for us since we are currently in the process of experimentally solving its solution structure. Via a BLAST [16] search for the primary sequence of Ppar *γ *we identified several related proteins for which three-dimensional structures are available (Table 2), namely Ppar *α *[42-44] (PDB codes: 1K7L, 1KKQ, and 1I7G) and Ppar *δ *[45] (1GWX and 3GWX).

**Figure 2:**
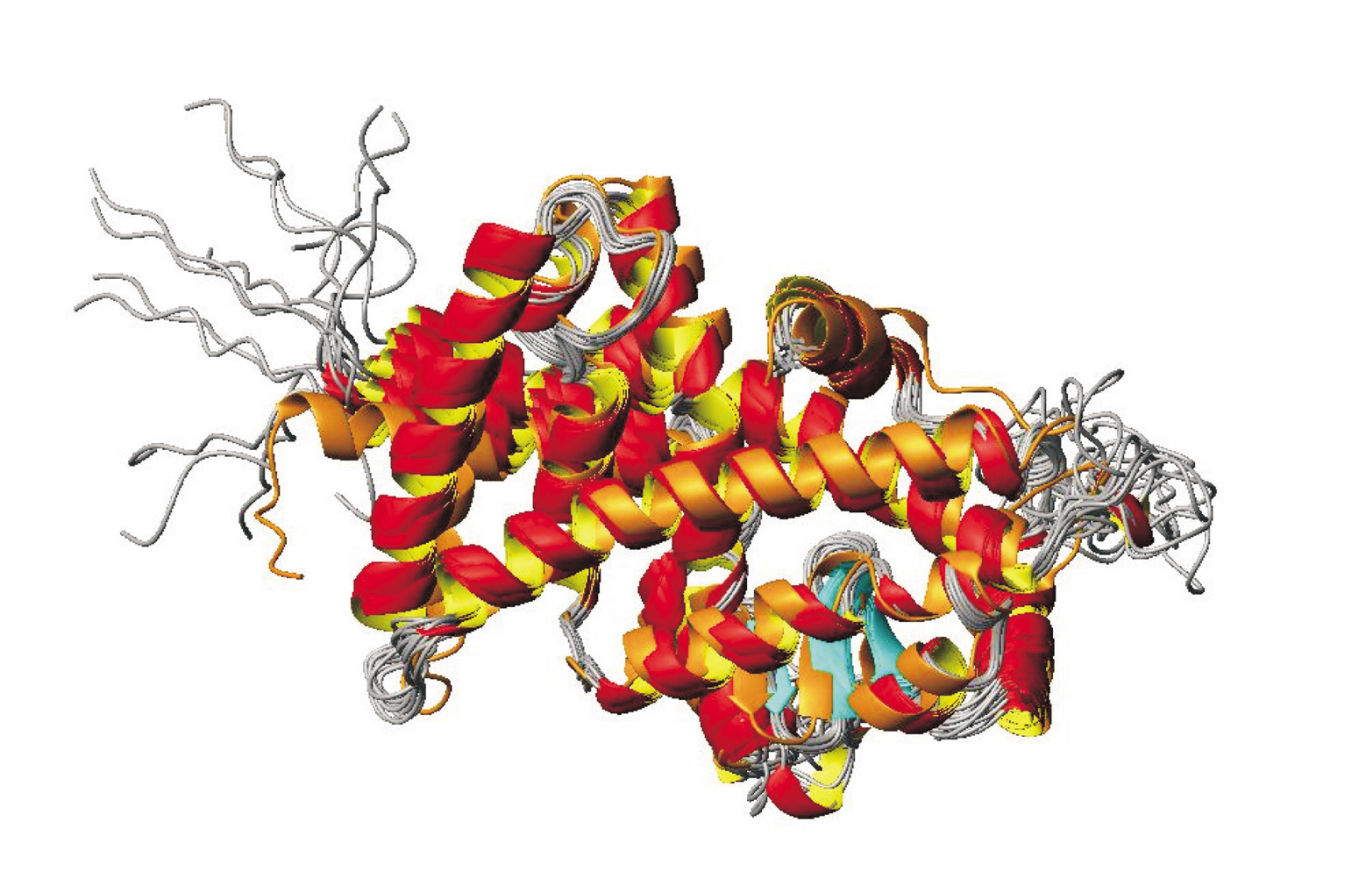
*Comparison of the model structure of Ppar γ from human with the corresponding X-ray structure*. Overall good agreement between the bundle of final model structures (helices in red and yellow, *β*-strands in blue and loops in grey) and the X-ray structure (orange) is obtained. Deviations are mainly seen in larger loop regions, the unstructured N-terminus and at the C-terminal end.

Model structures were calculated as detailed for HPr and out of 125 calculated structures the 16 structures with the lowest pseudo energies were further analyzed. A summary of the used restraints is given in Table 4. These sixteen models showed a good convergence with a RMSD value for the backbone atom positions of 0.135 nm (residues 206 – 477) (Fig. 2, Table 6). The secondary structure elements observed in the model structures agree well with the corresponding X-ray structure of the template protein, comprising a four-stranded antiparallel *β*-sheet and twelve *α*-helices (Fig. 2). Analysis of the ensemble of the selected sixteen structures with PROCHECK-NMR [41] showed that almost all backbone dihedral angles fell into the most favored and additionally allowed regions of the Ramachandran plot (Table 6).

### Comparison to target structures

**Figure 3:**
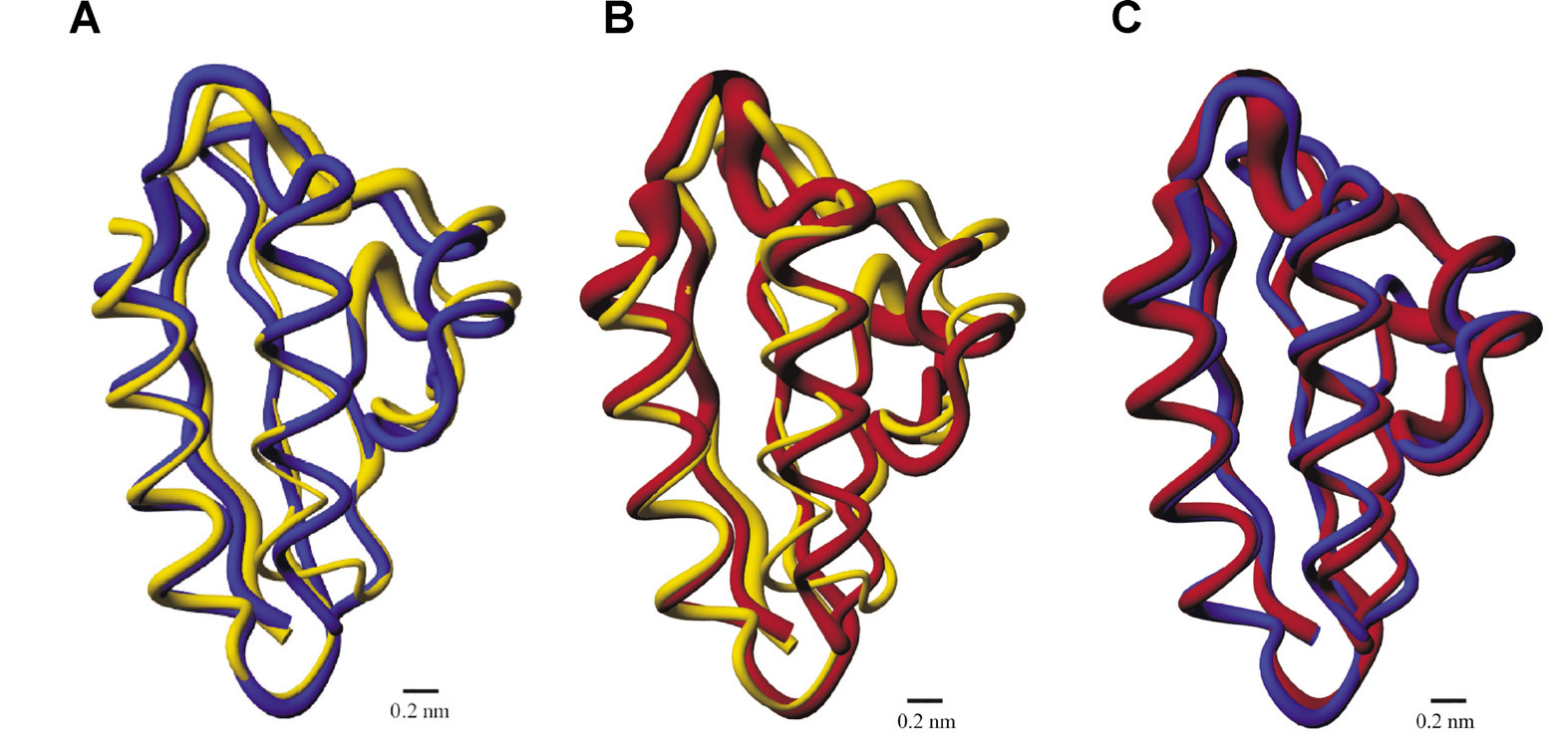
*Comparison of the model structure of HPr from E. coli with the corresponding X-ray and NMR structures*. A comparison of the modeled HPr homology structure with the structures experimentally determined by NMR spectroscopy (1HDN) and X-ray crystallography (1POH). The structures are shown in the same orientation as in Fig. 1 with the radius of the backbone splines indicating the RMSD of the C^*α *^atom positions in the respective structures. (A) Overall good agreement between the model structure (yellow) and the X-ray structure (blue) is obtained. Deviations are mainly seen in loop regions and in the orientation of helices a and b. RMSD values for the C^*α *^atom positions of the X-ray structure 1POH have been derived from the crystallographic B-factors, *f*_B_, using the Debye-Waller equation 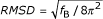 where isotropic displacement from the mean atom positions was assumed. (B) Comparison of the model (yellow) and the NMR structure (red). Deviations are seen in the same regions as before. (C) X-ray (blue) and NMR (red) structures superimpose well. Interestingly, deviations between them are mainly observed in regions where the two structures also diverge from the homology model.

The ensemble of modeled HPr structures was compared to the target structure of HPr from *E. coli *which before had been elucidated using NMR spectroscopy (1HDN) and X-ray crystallography (1POH). For 1HDN a bundle of 30 structures was deposited in the protein database. As stated in the header of the coordinate file the first structure is closest to the ensemble average. As a consequence this structure was selected as the NMR target structure. A comparison between the modeled structure and the target NMR and X-ray structures is shown in Fig. 3. The homology model displayed the same global fold and distribution of secondary structure elements as both target structures. To quantify the agreement between the individual structures the root mean square deviations (RMSD) between the different structures were calculated for the backbone atom positions. While the RMSD between the two target structures 1HDN and 1POH amounted to 0.11 nm the comparison of the best modeled structure with the target NMR structure and the X-ray structure yielded RMSD values of 0.17 nm and 0.15 nm, respectively. Although the agreement between the modeled and the target structures was worse than the agreement between the two target structures, the RMSD values were of similar magnitude. Deviations between the homology model and the experimentally determined structures were mainly seen in the loop regions and in the orientation of helices a and b. Interestingly, these are also the regions that are least well defined in the X-ray and NMR structures and where these structures diverge most. In contrast, the core region of HPr and its overall fold are reproduced well in the homology model.

Further, we used R-factor analysis [46] to compare the modeled structure to the target structures. The quality of the protein backbone was specifically assessed by only taking into account spectral signals arising from backbone protons. Low R-factors of similar magnitude were obtained when comparing the modeled structure with either the NMR target structure (R-factor 0.093) or the X-ray target structure (0.076). Consistent with the RMSD values the R-factors also indicated that the homology structure more closely resembled the X-ray structure than the NMR structure. A slightly lower R-factor of 0.073 was obtained when comparing the two target structures with each other (Table 7).

For Ppar *γ *the best model structure in terms of pseudo-energy was compared to the target X-ray structure (3PRG). The agreement between the two structures was assessed by calculating the corresponding RMSD value for the backbone atoms, which amounted to 0.262 nm (Table 8). Note that the first five unstructured residues and the region between residues 262 and 274 which were missing in the X-ray target structure were not considered in this analysis. Deviations between the homology model and the X-ray structure were mainly seen in the loop regions and in the orientation of the helices preceding and following the unstructured region between residues 262 and 274. The agreement between model and X-ray structure was further analyzed by the calculation of pseudo NMR R-factors (Table 8). Although somewhat higher R-factors were obtained for Ppar *γ *than for HPr, the R-factor analysis still showed a reasonable agreement between model and X-ray structure.

### Importance of torsion angles

**Figure 4:**
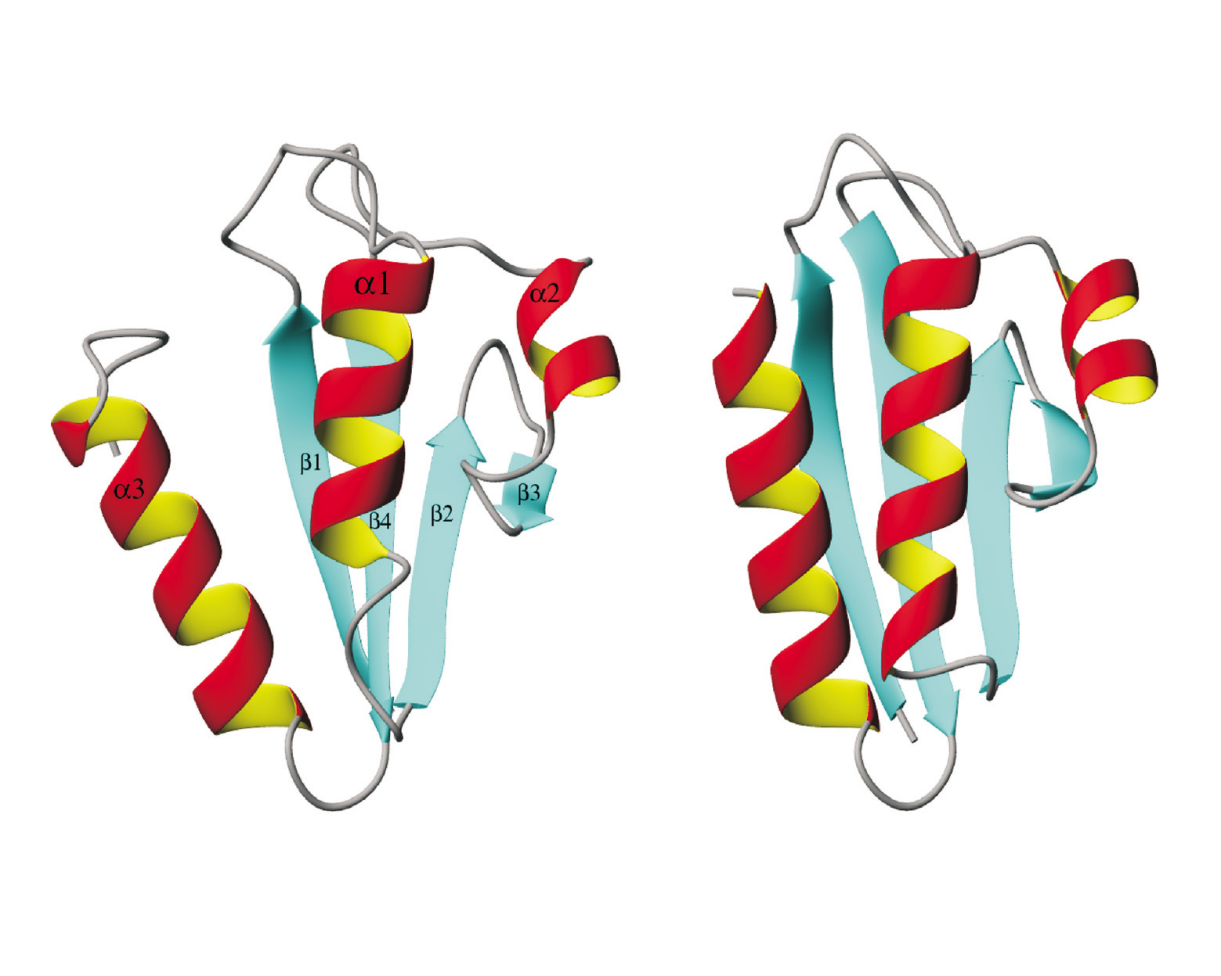
*Importance of torsion angle restraints exemplified on HPr from Streptococcus faecalis*. On the left hand side the model structure calculated with PERMOL using 427 torsion angle restraints and 41 hydrogen bonds is displayed, while on the right hand side the target X-ray structure 1PTF is shown. The RMSD value for the heavy atoms of the two structures is 0.328 nm. Restraints for torsion angles and hydrogen bonds were directly generated from the X-ray structure 1PTF.

In principle, torsion angles can completely define the 3D-structure of a protein when the general geometry of the amino acids is predefined. However, small errors of torsion angles in the backbone propagate and lead to large errors in the Cartesian space for amino acids remote in the sequence. Nevertheless, torsion angles are optimal predictors for local folding. Fig. 4 exemplifies the importance of the torsion angles for the structure predictions. As an example it shows a structure prediction (calculation) of HPr from *S. faecalis *from a rather small number of restraints created from the X-ray structure (1PTF) of the protein. Only 427 torsion angle restraints together with 41 hydrogen bond restraints can be sufficient to determine the various secondary structure elements together with the global fold of the molecule. Even the loop regions for which no hydrogen bond restraints are present adopt native-like conformations. Only the third *α*-helix is rotated away from the core of the protein since its orientation is solely defined by the angle restraints of residues 67–69.

## Discussion

In this contribution we have presented a new program for homology modeling of protein structures. Using restraint molecular dynamics simulations together with spatial restraints derived from template structures we calculated homology structures of HPr from *E. coli *and of human Ppar *γ*. An advantage of the proposed method is the use of spatial restraints with individual upper and lower limits depending on the local structural conservation in the template structures. This becomes especially evident for the obtained bundle of Ppar *γ *model structures where one can easily distinguish between the mostly well-defined secondary structure elements and less ordered regions e.g. some of the larger loop regions.

At first glance it appears to be a disadvantage of the proposed method that not a unique, seemingly perfect structure is the result of the calculations as in the case of threading methods. However, the structure bundle produced by our approach gives an idea of the conformational subspace determined by the available experimental basis and the physical model. This is a safeguard against typical over-interpretations of model structures where data in badly predictable regions are used for the detailed interpretation of functional data or are used during the drug design process.

An additional advantage of the simulated annealing approach is that restraint violations are not treated explicitly but contribute to the overall "energy" which is minimized. In contrast to other methods in the approach used in PERMOL the mean torsion angles and their errors provide the main information. A few distance restraints are used to define the long-range relations which cannot be described sufficiently well by the local data. Accordingly, details of the selection of these restraints are not critical. Thus, the selection of pairwise restraints between all conserved residues seems to be plausible. The same is true for conserved hydrogen bonds. However, the PERMOL software also allows to define a custom selection of restraints and thus an adaptation to specific needs. As an example all hydrophobic contacts between amino acid residues observed in the template structures could be selected to serve as restraints. The automated calculation of individual weighting factors during the calculation of the expectation values and standard errors of the individual restraints would permit to introduce information about the local and global sequence conservation and the precision of the used structures. Currently, we are undertaking efforts to address this question. The high quality of the structure models generated with PERMOL illustrates that the same MD programs used for the determination of NMR structures can also be utilized for homology modeling. The programs and strategies developed for NMR structure determination have evolved to efficient optimizers even when only limited information (i. e. small number of structural restraints) is available. This has been recognized for example by Dominguez *et al*. [47] who use restrained molecular dynamics together with the ARIA protocol [48] for solving the docking problem. While in the case of NMR structure determination the restraints that enter the molecular dynamics simulation are derived from experimental observables like NOE cross-peaks, *J-*couplings, and residual dipolar couplings, in the case of homology modeling synthetic restraints are generated from previously determined structures of homologous template proteins. The use of standard MD programs and protocols also has a disadvantage since it is not possible to directly introduce properties in the calculation which are not provided for by the programs. An example would be the use of specific potential forms with multiple minima which describe the homology-derived information in more detail as it is done e. g. by MODELLER [28].

We compared the HPr homology structure we obtained with PERMOL to a structural model of HPr from *E. coli *calculated using MODELLER (version 6v2). When the same alignment file and template structures were used, homology models of similar quality were obtained with the two programs.

A specific advantage of the approach presented here is that it can be well used in the context of standard structure determination by NMR. The restraint files generated by PERMOL are editable and can be easily combined with other data and be adapted for use with different programs. As the same MD programs are used both for modeling with PERMOL and for NMR structure determination, incomplete experimental data can be conveniently combined with spatial restraints derived from homologous template proteins. The validity of the resulting structure models can be checked by calculating NMR R-factors [46]. Different force fields and annealing protocols which are available for the NMR MD programs can also be utilized for homology modeling. In this way recent advances like the structure refinement in explicit solvent [49,50] can be readily exploited to derive more accurate homology structures.

## Conclusion

In summary, we have presented a new method for homology modeling capable of producing high-quality structure models. Compared to many other homology structure prediction programs it is based on a different philosophy since its aim is not to predict a unique best structure but a bundle of structures representing the locally different degrees of reliability of the structure prediction. Since the homology-derived restraints are mainly used to reduce the conformational space to be searched by the MD calculation, their relative importance for obtaining a correct homology model is expected to decrease in future time as the physical model employed in these calculations is improved. Another advantage of the approach described here is its flexibility, conveniently allowing several template structures to be included as sources of structural restraints. Furthermore, the PERMOL software permits to determine which kinds of structural restraints enter the molecular dynamics calculation in a controlled fashion. We demonstrated that the standard MD programs used in the course of structure determination by NMR can also be well utilized for the purpose of homology modeling. Prediction on the basis of averaged torsion angles is a powerful tool which efficiently makes use of the structural information available in the protein data base and leads to well-defined structures.

Recently, a homology model determined with PERMOL was used in the resonance assignment [51] and structure determination process of a mutant form of HPr from *S. carnosus *[52] and to obtain an initial estimate for the molecular alignment tensor describing the partial orientation of the HPr molecule in anisotropic solution [53,54]. PERMOL has also been integrated in the NMR structure determination package AUREMOL [39]. In this molecule-centered top-down approach one starts with a trial structure e.g. a homology model obtained by PERMOL that is iteratively refined until it fits the experimental data sufficiently as verified by the calculation of NMR R-factors.

## Methods

### Calculation of the restraints for simulated annealing

Structural information obtained from a set of homologous structures j (j = 1,..,N_i_) must be expressed in form of restraints. The restraint of a parameter *α*_i _is usually defined by its expectation value  and the upper and lower limits  and , respectively. PERMOL offers several ways to calculate these quantities from the expectation values observed in the template proteins <*α*_i_> and the corresponding standard deviations *s*_i_. For non-cyclic parameters <*α*_i_> and *s*_i _can be simply calculated according to eqs. (1) and (2).



and



with the weighting factor  for a given event *i *and the total number of events *N*_i_. For cyclic parameters like dihedral angles, which are mainly used within PERMOL such a definition does not directly apply but can be extended by the approach described by Döker et al. (1999) [34].

Here, the origin of the coordinate system is shifted to fulfill the condition



and the standard deviation is calculated according to eq. (2). The expectation value  is obtained by



The parameters  determine the statistical weight of a given homology structure used to calculate a restraint. In principle, their value will depend on factors such as the local and global sequence conservation and the quality of a structure, e. g. when comparing X-ray and NMR-structures.

### Implementation overview

In order to facilitate the determination of structural restraints for homology modeling the software package PERMOL was developed. PERMOL was written in Perl/Tk and has been tested with the operating systems SGI IRIX, Linux and Windows. The software and a detailed manual explaining its use can be obtained free of charge from the authors . Sequence alignment is done by using the program CLUSTALX [18]. Structure calculations are performed with output data files generated by PERMOL which can be imported by the molecular dynamics programs DYANA [32] and CNS [33]. Dihedral angles from different structures are averaged following the algorithm described by Döker *et al*. [34]. The typical computing time for setting up the restraint and parameter files for the MD-calculation is negligible using a modern PC. The calculation of the structures strongly depends on the MD-program used, the number of structures calculated and the actual simulated annealing protocol. In the examples presented here structures were calculated on a standard Linux-PC using the MD program DYANA. The corresponding calculation times for a single structure model were around 30 and 160 seconds for HPr and Ppar *γ*, respectively. Figures 1, 2, 3, and 4 have been prepared with MOLMOL and rendered with PovRay .

### Validation of homology models

Modeled structures can be quantitatively compared to their respective target structures by calculating NMR R-factors according to [46]. Analogous to crystallography R-factors, NMR R-factors are used to quantify how well a three-dimensional structure accounts for the spectral signals occurring in an experimental NMR spectrum. Using an implementation of the complete relaxation matrix analysis (RELAX, [56,57]) artificial NMR spectra are calculated for the given three-dimensional structure and compared to the experimental spectra. R-factors quantify the deviations between the two types of spectra and are therefore a measure for the quality of the trial structure. In the case of perfectly matching spectra the R-factor adopts a value of 0. Analogous, R-factor analysis can also be employed to quantify the agreement between two protein structures. In that case artificial NMR spectra are calculated for both structures and are compared to each other.

The agreement between two structures can be further assessed by determining the root mean square deviations (RMSD) between the atom positions of the structures. The program MOLMOL [55] is used to fit the structures atop of each other and to calculate RMSD values. The stereo-chemical quality of the obtained models was validated using the program PROCHECK-NMR [41].

## Abbreviations

HPr: histidine-containing phosphocarrier protein, MD: molecular dynamics, NMR: nuclear magnetic resonance, NOE: nuclear Overhauser effect, PDB: Protein Data Bank Brookhaven, Ppar *γ*: human peroxisome proliferator activated receptor *γ*, PTS: phosphoenolpyruvate carbohydrate phosphotransferase system, RMSD: root mean square deviation

## Authors' contributions

TM, WG, and HRK conceived the project. DW and TM performed initial feasibility studies and refined the overall modeling strategy. AM wrote the PERMOL software and a manual. AM, DW, and WG calculated the homology structures. AM drafted the manuscript. WG and HRK coordinated the study and wrote the manuscript. All authors read and approved the final manuscript.

## Figures and Tables

**Table 1:** Statistics of PDB structure files used for HPr

**PDB code**	**Organism**	**Method**	**Resolution [nm]**^a^	**Reference**
1HDN	*E. coli*	NMR	0.20	[36]
1POH	*E. coli*	X-ray	0.20	[37]
1PTF	*S. faecalis*	X-ray	0.16	[58]
1QFR	*E. faecalis*	NMR	0.27	[59]
1QR5	*S. carnosus*	NMR	0.28	[60]
2HID	*B. subtilis*	NMR	0.19	[61]

^a^The equivalent resolution of the NMR structures was calculated using PROCHECK-NMR [41].

**Table 2:** Statistics of PDB structure files used for Ppar *γ*

**PDB code**	**Organism**	**Method**	**Resolution [nm]**	**Reference**
3PRG	*human*	X-ray	0.29	[38]
1K7L	*human*	X-ray	0.25	[42]
1KKQ	*human*	X-ray	0.30	[43]
1I7G	*human*	X-ray	0.22	[44]
1GWX	*human*	X-ray	0.25	[45]
3GWX	*human*	X-ray	0.24	[45]

**Table 3:** Restraints for molecular dynamics calculation for HPr

**Type of restraint**	**Number**
inter-atomic distances	186
hydrogen bonds	50
backbone dihedral angles	164

**Table 4:** Restraints for molecular dynamics calculation for Ppar *γ*

**Type of restraint**	**Number**
inter-atomic distances	1391
hydrogen bonds	153
backbone dihedral angles	528

**Table 5:** Structural statistics for HPr

**RMSD values for the ten lowest-energy structures**	**RMSD [nm]**
backbone atoms C^*α*^, C', N	0.041
heavy atoms	0.111

**Residues in the Ramachandran plot**	**Incidence^a^**

most favored regions	87.2 %
additional allowed regions	12.8 %
generously allowed regions	0.0 %
disallowed regions	0.0 %

^a^The dihedral angles have been analyzed using the program PROCHECK-NMR.

**Table 6:** Structural statistics or Ppar *γ*

**RMSD values for the sixteen lowest-energy structures**	**RMSD [nm]**
backbone atoms C^*α*^, C', N	0.135
heavy atoms	0.191
	
**Residues in the Ramachandran plot**	**Incidence**^a^

most favored regions	84.1 %
additional allowed regions	14.3 %
generously allowed regions	1.4 %
disallowed regions	0.2 %

^a^The dihedral angles have been analyzed using the program PROCHECK-NMR.

**Table 7:** Comparison between model structures and experimental structures for HPr

**Structures**	**Quantities^a^**	**NMR target structure**	**X-ray target structure**
X-ray structure	backbone RMSD [nm]	0.106	0
	heavy atom RMSD [nm]	0.273	0
	R-factor	0.073	0

best NMR structure	backbone RMSD [nm]	0	0.106
	heavy atom RMSD [nm]	0	0.273
	R-factor	0	0.072

best model structure	backbone RMSD [nm]	0.169	0.147
	heavy atom RMSD [nm]	0.273	0.253
	R-factor	0.093	0.076

model structure bundle	backbone RMSD [nm]	0.178	0.154
	heavy atom RMSD [nm]	0.277	0.258
	R-factor	0.097	0.081

^a^Backbone RMSDs include N^H^, C^*α*^, and C' atoms. Heavy atoms include all atoms except protons. RMSDs are pairwise RMSDs. R-factors are calculated using the R-factor *R*_3 _according to [46] including only signals arising from backbone protons.

**Table 8:** Comparison between model structures and experimental structures for Ppar *γ*

**Structures**	**Quantities^a^**	**X-ray target structure**
best model structure	backbone RMSD [nm]	0.262
	heavy atom RMSD [nm]	0.317
	R-factor	0.260

model structure bundle	backbone RMSD [nm]	0.299
	heavy atom RMSD [nm]	0.355
	R-factor	0.231

^a^Backbone RMSDs include N^H^, C^*α*^, and C' atoms. Heavy atoms include all atoms except protons. RMSDs are pairwise RMSDs. R-factors are calculated using the R-factor R_3 _according to [46] including only signals arising from backbone protons.
